# Economic impact of disease progression in follicular non-Hodgkin lymphoma

**DOI:** 10.3109/10428194.2011.592623

**Published:** 2011-07-12

**Authors:** Roy Beveridge, Sacha Satram-Hoang, Kavita Sail, Joseph Darragh, Clara Chen, Michael Forsyth, Carolina Reyes

**Affiliations:** 1 Healthcare Informatics, US Oncology, Houston, TX, USA; 2 Genentech, Inc., South San Francisco, CA, USA

**Keywords:** Health resource utilization, costs of care, disease progression, follicular lymphoma

## Abstract

Using a retrospective claims database, we estimated the economic costs of progression among patients with follicular non-Hodgkin lymphoma (f-NHL) treated in an outpatient community-based setting. Patients with f-NHL who received care between 1 July 2006 and 31 December 2009 were categorized into two cohorts based on whether they experienced progressive disease (PD) or not. Costs per patient per month (PPPM) were compared between patients with PD versus non-PD. Follow-up time was censored at the last entry for disease status or 6 months after the date of remission/stable disease or progression. Of the 1002 patients with f-NHL identified, 268 progressed and 734 did not. The mean overall costs PPPM over the 6-month follow-up period were significantly higher for patients with PD versus non-PD ($3527 vs. $860; difference = $2667; *p* < 0.001). This cost difference persisted within all resource categories evaluated. Results of this study indicate that therapies which delay progression for patients with f-NHL may result in potential cost savings.

## Introduction

Follicular non-Hodgkin lymphoma (f-NHL) is a slow-growing (indolent) subtype of NHL that constitutes approximately 20-25% of all NHL and 70% of indolent lymphomas [1]. While f-NHL is considered incurable with currently available therapies, the 5-year survival rate is around 70% [2] with median survival being 8-10 years [3,4]. Often patients are not treated when first diagnosed if no symptoms are present. The natural history of f-NHL is characterized by continuous risk of relapse and progression, with each event becoming less sensitive to treatment and each remission shorter than the preceding one [5,6]. This makes disease management challenging, with a wide array of treatment options ranging from watchful waiting to intensive therapies that are typically aimed at delaying disease relapse and progression with fewest adverse effects.

The safety and effectiveness of rituximab (an anti-CD20 antibody) resulted in the widespread use of this agent in the treatment of f-NHL as either monotherapy or in combination with chemotherapy [7,8]. Rituximab added to chemotherapy has demonstrated significant increases in response rates, response duration, and overall survival as either first-line or relapse treatment in NHL [9-12]. Consolidation therapy with rituximab followed by ^90^Y-ibritumomab tiuxetan in patients with f-NHL who achieved complete or partial response after first-line induction therapy prolonged progression-free survival (PFS) [13]. The use of maintenance rituximab after chemotherapy has been shown to be superior to observation among patients with NHL not previously treated with rituximab [14,15]. Further, maintenance rituximab was beneficial in patients with f-NHL who achieved a partial or complete remission after initial single-agent first-line rituximab therapy [16,17]. Maintenance rituximab significantly improved PFS compared with observation after induction with both chemotherapy alone and rituximab plus chemotherapy in relapsed/resistant f-NHL [18,19]. Most recently, data presented from a large international phase III trial demonstrated that patients with advanced f-NHL treated with rituximab maintenance had a 50% reduction in risk of progressive disease (PD) relative to patients who did not receive rituximab maintenance [20,21].

While current treatments have demonstrated improved clinical outcomes among patients with f-NHL, growing constraints on healthcare resources are making it increasingly important to also evaluate economic outcomes associated with therapy. Several studies have compared the cost-effectiveness of treatment alternatives, and found that the use of rituxumab is cost-effective in the first-line [22] and relapsed maintenance settings [23]. Kutikova *et al*., in a study evaluating medical costs associated with NHL in the first 2 years of treatment, found that treatment failure was the most expensive clinical scenario [24]. Past economics studies, however, are limited in geographic range and are often restricted to the clinical trial setting. Furthermore, no studies have directly evaluated the economic consequences of PD following a positive treatment response.

The goal of this study was to quantify the incremental cost of PD and associated healthcare resource utilization (HRU) among patients with f-NHL following successful first-line therapy within a large geographically dispersed network of community-based outpatient oncology practices in the United States. Results of this study may help to quantify the economic benefits of delaying progression of f-NHL in the real-world setting.

## Materials and methods

### Data sources

This study utilized clinical data from US Oncology's iKnowMed oncology-specific electronic medical record (EMR) system. This system captures demographic, clinical, and treatment data for patients receiving care within US Oncology's network of approximately 1200 community-based oncologists. During the study time period, the iKnowMed EMR system was implemented across approximately 82% of the US Oncology network. It is estimated that iKnowMed captures data on approximately 5-6% of all newly diagnosed patients with NHL in the United States, in a setting where patients are treated according to usual clinical practice with no criteria for therapy selection and no schedule of visits imposed.

To estimate outpatient cost of care, we linked patients with NHL identified in the iKnowMed EMR to US Oncology's Claims Data Warehouse (CDW). The CDW repository houses all claims for services provided within the US Oncology network. Data include HCPCS/CPT (Healthcare Common Procedure Coding System/Current Procedural Terminology) codes and descriptions, date of service, quantity, amount billed, and primary payer. Data were de-identified and accessed in compliance with the Health Insurance Portability and Accountability Act. Therefore, approval by an institutional review board was not required.

### Study population

Using a retrospective cohort design, we identified a pool of 2005 patients with f-NHL in iKnowMed who achieved partial or complete remission or had documented stable disease or progression from 1 July 2006 to 30 December 2009. Treatment response and PD were classified according to standard response criteria [25]. Patients were categorized into two cohorts depending on their experience of PD during the study catchment period. Incidence of PD was identified by a documented disease status of 'progressive disease’ following a period of remission or stable disease. Patients who did not progress were identified by a documented disease status of ‘partial remission,’ ‘complete remission,’ or ‘stable disease.'

There were 282 (14%) patients with PD and 1723 (86%) without. Of these, we linked 1865 (93%) to the CDW. We excluded 36 patients (five with PD and 31 without PD) who were enrolled in another clinical trial or received treatment for another cancer, and 848 patients (nine with PD and 839 without PD) who were identified as having second opinion/ consult only. The identification of second opinion/consult only cases was based on having fewer than 30 days of total follow-up in iKnowMed with incomplete demographic and clinical characteristics. After applying the inclusion and exclusion criteria, a total of 1002 patients with f-NHL were included in the final study population: 734 in the ‘no PD’ cohort and 268 patients in the ‘PD’ cohort.

Follow-up time was censored at the last entry for disease status, end of 12 months, or study end (31 December 2010). To calculate the cost of progression, we adopted a phase of care approach using a 6-month window from the date of disease progression, as the majority of re-treatment costs are usually seen within this time period [26-28]. The study index date (baseline) was the date of PD or the initial date in the study catchment period for those with no PD. Data were collected at baseline on patient demographics (age at diagnosis and gender), clinical characteristics (stage at diagnosis, presence of B-symptoms, hemoglobin [HGB] levels, lactate dehydrogenase [LDH] levels, performance status, and nodal status) as well as payer type. The number of lymph nodes and extent of spread to the lymph nodes is captured in the iKnowMed data based on the TNM staging, which captures the extent of the tumor (T), extent of spread to the lymph nodes (N), and presence of distant metastasis (M). For laboratory values, performance status, and nodal status, we captured the closest entry within 14 days of the index date.

### Statistical analysis

Patients were described at baseline with respect to demographic and clinical characteristics overall and stratified by progression status. χ^2^ tests for categorical variables and *t*-tests for continuous variables determined statistically significant differences by progression status.

Costs were estimated based on unadjusted 2007 Medicare reimbursement rates, Geographic Practice Cost Index (GPCI) 93. While reimbursement rates were available for the large majority (97%) of charges, for the remaining charges missing a Medicare rate we imputed costs using a median charge-to-cost ratio that was calculated using all codes for which Medicare reimbursement was available. Costs were calculated using a standard cost per patient per month (PPPM) metric. For months in which a patient did not accrue costs, a value of zero was applied to ensure that those patient-months were included in the denominator for the costs PPPM calculations. Costs were reported overall and by health resource category. Unadjusted costs were compared between PD cohorts using the Mann-Whitney *U*-test.

We developed econometric models to adjust for clinical and demographic differences that may confound the observed association between progression status and cost of care. A backward elimination approach was utilized to identify significant predictors of total costs. Age at diagnosis, gender, stage at diagnosis, presence of B-symptoms, baseline HGB, baseline LDH, number of positive lymph nodes, and baseline performance status were considered as possible covariates. PD was included as an indicator variable. Standard model diagnostics was carried out including tests for multicollinearity and heteroscedasticity, and appropriate estimation methods were employed (e.g. calculating heteroscedasticity-robust standard error [SE]) [29].

To provide a more comprehensive evaluation of the extent of HRU for patients who did and did not progress, we compared the frequency at which patients received care over a 6-month period. Using claims data, we compared the number of billed outpatient physician visits, acute care visits (inpatient and emergency room [ER] admissions), outpatient chemotherapy infusion visits, and outpatient laboratory procedures by progression status. These frequencies were measured on a PPPM basis and Mests/Mann-Whitney *U*-tests (depending on the normality of data) were used to determine statistical significance. Statistical analyses were conducted with SPSS version 15 and SAS version 9.1. All statistical tests were interpreted at α = 0.05, two-tailed.

## Results

Table I shows the baseline clinical and demographic characteristics of patients overall and by progression status. The overall median age at diagnosis was 61 years and 36% of patients were older than 65. There were no significant differences in age at diagnosis and gender between PD and non-PD patients. Overall 44% of patients were male and 56% were female. While there was no significant difference in the percentage of patients presenting with B-symptoms at diagnosis, patients who progressed were more likely to present with advanced stage at diagnosis compared to non-PD patients (41% vs. 32%; p = 0.06). Lymph node status, baseline (measured at index date) laboratory values (HGB and LDH), and performance status were significantly worse for patients with PD compared to non-PD. The vast majority of patients were covered by Medicare (53%) or had private insurance (44%).

**Table I:** Patient characteristics at baseline.

Characteristic	Total (*n* = 1002)	No progression (*n* = 734)	Progression (*n* = 268)	*p*-Value
Age at diagnosis, *n* (%)				
<55	333 (33)	258 (35)	75 (28)	0.10
55-65	313 (31)	222 (30)	91 (34)	
>65	356 (36)	254 (35)	102 (38)	
Mean age	60.1	59.6	61.3	
Median age (range)	61 (21-91)	60 (21-91)	61 (27-90)	0.10
Gender, *n* (%)				
Female	565 (56)	430 (59)	135 (50)	0.02
Male	437 (44)	304 (41)	133 (50)	
Stage, *n* (%)				
I	187 (20)	147 (21)	40 (16)	0.06
II	198 (21)	150 (22)	48 (19)	
III	234 (25)	170 (25)	64 (25)	
IV	327 (35)	223 (32)	104(41)	
Missing	56	44	12	
B-symptoms, *n* (%)				
No	812 (85)	601 (87)	211 (82)	0.11
Yes	138 (15)	93 (13)	45 (18)	
Missing	52	40	12	
HGB < 12*, *n* (%)				
No	668 (81)	502 (85)	166 (73)	< 0.0001
Yes	152 (19)	90(15)	62 (27)	
Missing	182	142	40	
Elevated LDH*, *n* (%)				
No	477 (86)	353 (89)	124 (80)	0.01
Yes	76 (14)	45(11)	31 (20)	
Missing	449	336	113	
4 + positive nodes, *n* (%)				
No	543 (67)	412 (70)	131 (58)	0.0008
Yes	272 (33)	176 (30)	96 (42)	
Missing	187	146	41	
ECOG PS*, *n* (%)				
0	581 (68)	445 (71)	136 (60)	0.0005
1	209 (25)	145 (23)	64 (28)	
2 +	60(7)	33(5)	27(12)	
Missing	152	111	41	
Payer type				
Private	438 (44)	330 (45)	108 (40)	0.06*
Medicare	537 (53)	381 (52)	156 (58)	
Medicaid	9(1)	6(1)	3(1)	
Other	18(2)	17(2)	1(0)	
Follow-up time, *n* (%)				
< 12 months	56(6)	28(4)	30(11)	
≥ 12 months	938 (94)	700 (96)	236 (89)	
Mean follow-up time, months	35.1	37.0	29.8	
Median follow-up, months (range)	37(1-53)	39 (3-53)	32(1-53)	
Alive at end of follow-up, *n* (%)	983 (99)	725 (99.6)	258 (97)	

HGB, hemoglobin; LDH, lactate dehydrogenase; ECOG PS, Eastern Cooperative Oncology Group Performance Status.

* Based on entry in iKnowMed closest to index date (±14 days).

The majority of patients with PD (*n* = 186; 69%) received post-progression infusion therapy (Table II). Of these patients, 69 (37%) received rituximab monotherapy, 110 (59%) received rituximab in combination with chemotherapy, and seven (4%) received chemotherapy only. Among the non-PD group, 195 (27%) received infusion therapy. The majority of these patients (re = 147; 75%) received rituximab monotherapy, while 44 (23%) received rituximab in combination with chemotherapy and four (2%) received chemotherapy alone. To further verify why some non-PD patients received chemotherapy other than maintenance rituximab, we conducted an electronic chart review of a random sample of this group and found that chemotherapy administered was typically given to complete a planned chemotherapy regimen following treatment response (i.e. consolidation). For example, the typical non-PD patient who had chemotherapy and rituximab charges actually only received chemotherapy for a short time following treatment response and then subsequently received rituximab maintenance therapy.

**Table II:** Description of infusion therapy by progression status after index date.

Infused therapy	Total (*n* = 1002)	No progression (*n* = 734)	Progression (*n* = 268)
Infusion therapy			
No	621 (61.9%)	539 (73.4%)	82 (30.5%)
Yes	381 (38.1%)	195(26.5%)	186 (69.4%)
Rituximab monotherapy	216(21.6%)	147(75.4%)	69(37.1%)
Rituximab-chemotherapy	154 (15.4%)	44(22.5%)	110(59.1%)
Chemotherapy only	11 (1.1%)	4(2.1%)	7(3.8%)
Chemotherapy agents			
Rituximab-chemotherapy	154	44	110
Cyclophosphamide	108	33	75
Vincristine	101	32	69
Doxorubicin	41	16	25
Fludarabine	23	3	20
Other	203	46	157
Chemotherapy only	11	4	7
Cyclophosphamide	6	2	4
Vincristine	4	1	3
Doxorubicin	2	1	1
Etoposide	1	1	0
Other	10	2	0

**Figure 1:**
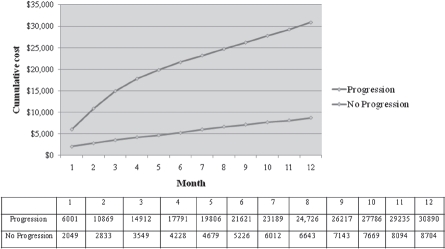
Cumulative 12-month total cost by progression status.

Figure 1 shows the cumulative total cost over 12 months by progression status. The cumulative 6-month total cost for patients with PD was $21 621 vs. $5226 for non-PD patients. At 12-months, the cumulative total cost for patients with PD was $30 890 vs. $8704 for non-PD patients. Table III presents a comparison of mean costs PPPM by progression status. Average costs PPPM over the 6-month follow-up period for patients with PD were $2667 more than for non-PD patients (*p* < 0.001), with a relative cost four times higher. Differences in cost were significant in all categories.

**Table III:** Six-month mean cost per patient per month overall and by category.

Service	No progression (*n* = 734)	Progression (*n* = 268)	C_p_–C_NP_	C_p_/C_NP_	*p*-Value*
Overall cost	859.98	3527.4	2667.4	4.10	<0.001
Outpatient visits	36.68	99.92	63.24	2.72	<0.001
Acute care†	2.46	24.14	21.68	9.81	<0.001
Chemotherapy	655.75	2495.01	1839.26	3.8	<0.001
R-mono	487.07	849.50	362.43	1.74	<0.001
R-chemo	166.07	1610.86	1444.79	9.69	<0.001
Chemo	2.61	34.65	32.04	13.27	<0.001
Other medication	101.86	700.28	598.42	6.87	<0.001
Laboratories	11.35	28.53	17.18	2.51	<0.001
Minor procedures	2.79	8.79	6.00	3.15	<0.001
Other	0.59	3.02	2.43	5.11	<0.001
XRT	15.07	71.27	38.93	4.72	<0.001
RAD non-XRT	32.59	95.41	62.82	2.92	<0.001

C_p_, cost of progression; C_Np_, cost of not progressing; R-mono, rituximab monotherapy; R-chemo, rituximab-chemotherapy; Chemo, chemotherapy only; XRT, external radiation treatment; RAD non-XRT, radiation other than XRT.

* Mann-Whitney *U*-test.

† lnpatient and ER visits.

In a level-level multivariable regression model (Table IV) adjusting for baseline hemoglobin level, number of positive lymph nodes at diagnosis, baseline performance status, and progression status remained significantly associated with total outpatient costs. PD was associated with an increased cost of $2557 per month, all else being equal. Due to the presence of heteroscedasticity as diagnosed using the Breusch-Pagan test, this model includes robust-SEs. In another model including log-transformed cost as the dependent variable (Table IV), PD was found to be independently associated with a two-fold higher cost after adjusting for potential confounders.

**Table IV:** Multivariable regression analysis of mean cost per patient per month.

	Untransformed model			Log-transformed model		
		
Covariate	Coefficient	95% CI	*p*-Value	Coefficient	95% CI	*p*-Value
Progression status						
No	Referent			Referent		
Yes	2557.20	2090.90-3023.49	<0.001	2.29	1.94-2.64	<0.001
Hemoglobin < 12						
No	Referent			Referent		
Yes	783.65	283.73-1283.57	0.002	1.01	0.64-1.38	<0.001
4+ positive nodes						
No	Referent			Referent		
Yes	558.92	166.99-950.84	0.005	0.62	0.28-0.95	<0.001
ECOGPS						
0	Referent			Referent		
1	611.48	180.07-1042.89	0.006	0.65	0.28-1.02	0.001
2	168.26	602.06-938.60	0.66	0.21	0.36-0.80	0.46

CI, confidence interval; ECOG PS, Eastern Cooperative Oncology Group Performance Status.

Table V presents health resource utilization by progression status. Patients who experienced PD had a 23% higher frequency of outpatient physician visits than non-PD patients (*p*< 0.001). A two-fold higher frequency was observed for outpatient laboratory visits (*p*< 0.001) in PD vs. non-PD patients. Patients with PD were significantly more likely to receive chemotherapy than non-PD patients (72% vs. 29%, respectively; *p*< 0.001). Further, among patients who received chemotherapy, those who progressed had a significantly higher frequency of chemotherapy infusion visits *(p <* 0.001), suggesting that patients who progress receive more intensive chemotherapy regimens than those who do not progress (the majority of whom receive maintenance rituximab). Similarly, patients with PD were significantly more likely to have an inpatient admission or ER visit than non-PD patients (18% vs. 4%; *p <* 0.001), although the mean number of acute care visits PPPM did not differ by progression status.

**Table V:** Comparison of healthcare resource utilization by progression status.

Service	No progression (*n* = 798)	Progression (*n* = 204)	*p*-Value
Chemotherapy visits			
No	530 (72.2%)	77 (28.7%)	<0.001
Yes	204 (28.8%)	191 (72.3%)	
Visits per patient-month*			
Mean	0.17	0.88	<0.001
Median (range)	0.16(0-2.84)	0.66(0-5)	<0.001
Outpatient physician visits			
Visits per patient-month			
Mean	0.47	1.23	<0.001
Median (range)	0.33 (0.16-7)	1 (0.16-9.33)	<0.001
Outpatient laboratory procedures			
Procedures per patient-month			
Mean	0.99	2.46	<0.001
Median (range)	0.66(0-15.6)	1.5(0-16)	<0.001
Acute care visits			
No	703 (95.7%)	220 (82%)	<0.001
Yes	31 (4.3%)	48 (18%)	
Visits per patient-month†			
Mean	1.11	1.72	0.62
Median (range)	0.66(0.16-3.33)	0.66(0.16-6.5)	0.62

* Of patients with at least one chemotherapy visit.

† Of patients with at least one acute care visit.

## Discussion

For a relatively indolent cancer such as f-NHL, for which treatment options range from watchful waiting to costly stem-cell transplant, rational treatment selection should include consideration of both the medical efficacy and the economic outcomes of the available treatment options. Results of this retrospective study highlight and quantify the economic costs of progression among patients with f-NHL treated within an outpatient community-based setting. Using linked EMR and claims data from a large cohort of patients, we provide further evidence to support the hypothesis that treatment strategies that delay or prevent progression not only improve clinical outcomes but also provide substantial economic benefits in lowering the costs of care in NHL.

Findings from this study add to the literature in that it is the first study to estimate the cost of progressed f-NHL and the burden of progression on the healthcare system. The few studies that have assessed the economic burden of NHL are subject to the following limitations: restricted to specific geographic regions, clinical trial setting, or a single employer; did not differentiate between NHL subtypes; and did not differentiate between treatment phase or progression status [30-32]. A recent study by Kutikova and colleagues evaluated medical costs by NHL subtype (indolent and aggressive), but examined costs of progression only for patients with aggressive NHL [24]. The current analysis accounts for these limitations and provides data for the cost of progression in a diverse group of patients with indolent NHL.

The present study showed that patients with f-NHL who progressed were more likely to have been diagnosed with advanced disease, have four or more positive lymph nodes, poor performance status, and high LDH and low HGB levels, consistent with other studies [33-35].

The overall crude cumulative total cost of progression over a 12-month follow-up period was $30 890, compared to $8704 for non-PD patients. The mean cost PPPM among those who progressed was $2667 more than for patients who did not progress (*p* < 0.001), with a relative cost nearly four times higher. In multivariate analysis, PD remained significantly associated with a two-fold higher cost, adjusting for differences in clinical factors. There are few studies of NHL costs with which to compare the results of this present study. Further, different patient populations, costing methodologies, and specific objectives make direct comparisons across studies difficult. In the only published US study of direct per-patient costs for f-NHL, Gleeson *et al.* estimated that the total direct costs for f-NHL in the first year following diagnosis was $36 000 [36]. In another study, the mean cost of treatment failure in aggressive NHL was $14 174 PPPM [24], which is significantly higher than the cost of progression estimated among patients with f-NHL in this study. However, different subsets of the patient population with NHL (aggressive vs. indolent) were considered, and while our study focused solely on outpatient costs, the study by Kutikova *et al.* considered inpatient costs as well [24].

We found that patients who progressed had significantly higher frequencies of outpatient physician visits, laboratory procedures, acute care visits, and intensive chemotherapy regimens compared to non-PD patients. This finding is supported by results from Kutikova *et al.* who showed that patients with both aggressive and indolent NHL had significantly higher resource use than controls [24].

In interpreting the findings, several factors need to be considered. First, although data were collected from geographically dispersed community oncology practices, it is subject to selection bias due to convenience sampling where the study population may differ in unknown ways from the underlying patient population. Second, potential confounding variables such as race, ethnicity, and income were not available, which limited the number of covariates that could be included in the multivariate analysis. We followed patients for a maximum of 6 months, which may have led to an underestimation of reported costs and the rate of treatment failure or PD. While the claims data used to estimate costs for this analysis included primarily outpatient medical oncology services, we also captured charges for radiologic services and inpatient consultation visits. However, costs for these services may be underestimated due to the possibility that patients may have received these outside of the US Oncology network.

However, despite these limitations, this study provides new information regarding the economic impact of PD in patients with f-NHL. As survival improves, the number of patients living with the disease is expected to rise. Patients whose disease has progressed may still have a relatively long survival with conventional management [33]. As a result, physicians will continue to navigate an increasing array of treatment options for this clinically heterogeneous group of patients. A better understanding of the economic outcomes associated with the disease and its treatment is essential in minimizing the burden to patients, caregivers, and society. Furthermore, while there is increasing scrutiny of the direct costs of prolonged treatment with targeted therapy, it is important to weigh these costs relative to the potential economic benefit of delaying progression. In this study, we objectively quantified the cost of PD, which plausibly could be offset by therapies that, while costly, have been demonstrated to yield significant clinical benefit in terms of delayed progression.
